# Enhancing quality of life in older adults: A comparison of muscular strength and power training

**DOI:** 10.1186/1477-7525-6-45

**Published:** 2008-06-13

**Authors:** Jeffrey A Katula, W Jack Rejeski, Anthony P Marsh

**Affiliations:** 1 Department of Health & Exercise Science, Wake Forest University, Winston-Salem, NC, USA

## Abstract

**Background:**

Although progressive resistance strength training (ST) has been found to improve various measures of physical functioning in older adults, the benefit to quality of life is unclear. Additionally, recent evidence suggests that high velocity power training (PT) may be more beneficial for physical functioning than ST, but it is not known whether this type of training impacts quality of life. The purpose of this study was to compare changes in multiple measures of quality of life resulting from ST vs. PT in older adults. A no exercise group was also included as control comparison condition.

**Methods:**

Forty-five older adults (M age = 74.8 years; SD = 5.7) were randomly assigned to either a) PT, b) ST, or c) control group (no exercise). Measures of self-efficacy (SE), satisfaction with physical function (SPF), and the Satisfaction with Life Scale (SWL) were assessed at baseline and following training. The resistance training conditions met 3 times per week for 12 weeks at an intensity of 70% 1 repetition maximum.

**Results:**

A series of ANCOVA's comparing between group differences in change and controlling for baseline values revealed significant group differences in all three measures: SE (F_(2,31) _= 9.77; p < .001); SPF (F_(2,32) _= 3.36; p = .047); SWL (F_(2,31) _= 4.76; p = .016). Follow up analyses indicated that the PT group reported significantly more change in SE, SPF, and SWL than the control group whereas the ST group reported greater change than the control group only in SE.

**Conclusion:**

These pilot data indicate that high velocity power training may influence multiple levels of quality of life over and above the benefits gained through traditional strength training.

## Background

Sarcopenia, the loss of skeletal muscle mass <2 standard deviations below the mean for young healthy adults [1], is a significant public health issue that affects approximately 45% of the older U.S. population [2]. It is a major contributing factor to the disablement process in that sarcopenia has been linked to gait and balance problems, increased fall risk, and the loss of functional independence [3,4]. Furthermore, advanced muscle loss in aging is related to the need for health support services, long-term care [1,5], and a reduced quality of life [6]. In fact, in the year 2000, the direct health care costs of sarcopenia among older adults was 18.2 billion dollars, which represented about 1.5% of total healthcare expenditures for that year [7].

Although sarcopenia has been found to be related to increasing age, it is not an inevitable component of the aging process and several risk factors are modifiable. Research indicates that sarcopenia is associated with lung disease [1], hormone levels [6], inadequate dietary protein intake [8], inflammation (measured by IL-6 and other cytokines) [4], and physical activity. Indeed, several randomized controlled trials have demonstrated that strength training (ST) can safely increase muscle mass and strength in older adults and improve physical function [for reviews see [9,10]]. Additionally, recent evidence [11] suggests that high velocity resistance training, or power training (PT), can result in significantly greater increases in muscle power compared to traditional ST and can significantly improve mobility related outcomes [12].

However, despite the well documented benefits of strength training on physical function, evidence examining the influence of ST on quality of life or quality of life in older adults is equivocal. For example, one study of cardiac rehabilitation patients (n = 38; M age = 59 years; SD = 12) found that twelve weeks of high intensity ST significantly improved several indices of quality of life, including self-efficacy, mood, physical function, pain, vitality, and role emotional health [13]. In contrast, Chin and colleagues (n = 173; M age = 81 years; SD = 5.4) [14] and Damush and Damush (n = 62; M age = 68 years; SD = 5.6) [15] found ST to have no effect on quality of life in older adults. Moreover, a recent comprehensive review of 62 trials examining the effects of ST on physical disability in older adults concluded that ST had no influence on pain, a global index of health-related quality of life, or vitality [9]. To our knowledge, there are no existing data concerning the effects of PT on quality of life.

Therefore, the purpose of this study was to compare the effects of ST and PT to one another and to a wait list control group with respect to changes in quality of life in older adults. Following Stewart and King's [16] conceptual framework of quality of life (QOL), we view QOL as consisting of both function and well-being. Furthermore, well-being consists of both proximal outcomes of physical activity as well as more global constructs [17,18]. Therefore, consistent with recommendations of Diener [19] and Rejeski and Mihalko [18], we chose to conceptualize QOL as consisting of multiple components of varying levels of specificity: satisfaction at both the global level (i.e., satisfaction with life) and domain level (i.e., satisfaction with physical function). We also chose to include a situation specific measure of self-efficacy as this construct has been found to be an important predictor of satisfaction with life [17] and to gauge activity-specific effects. Therefore, in this conceptual model one would expect resistance training to impact proximal, activity specific measures (self-efficacy), measures specific to functional domain (satisfaction with physical function), and global well-being (satisfaction with life).

## Methods

### Design

This study was a randomized, controlled 12-week trial that involved three different treatment conditions: PT, ST, or a wait-list control group. Older adults were randomly assigned to one of the three study arms following screening and baseline testing. The outcomes for this study included a global measure of quality of life, the satisfaction with life scale [20], a satisfaction index specific to physical functioning [21], and a self-efficacy measure that assessed perceived capability to lift different amounts of weight [22]. The present study was part of a larger pilot study examining the influence of ST vs. PT on muscle strength, muscle power, and physical functioning.

### Participants

Older adults were recruited from the local community using flyers distributed to retirement communities, a database containing the names of older adults interested in clinical trials research, and advertisements placed in the local newspaper. We were primarily interested in targeting older adults with impaired lower extremity functioning. Therefore, an inclusion criterion included self-reported difficulty with at least one of the following: walking a quarter mile, climbing stairs, getting in and out of a car, rising from a chair, lifting and carrying groceries, getting out of bed, getting out of the bathtub, or performing shopping, cleaning, or self-care activities. Additional eligibility criteria for participation were: age 65 years or older, community dwelling, and capable of walking unassisted or with a cane. Respondents were excluded if they were moving from the area within one year, under hospice care, receiving active treatment for cancer (other than skin cancer), had shortness of breath or chest pain at rest, myocardial infarction in the past 6 months, had a MMSE [23] score < 24, upper or lower extremity amputation, unstable CVD, severe CHF, stroke, peripheral vascular disease, CAD, valvular heart disease, major psychiatric disease (self-reported controlled depression was not an exclusion), severe anemia, liver or renal disease, uncontrolled diabetes or hypertension, orthopedic impairment, blindness or deafness, upper or lower extremity fracture within the past 6 months, consumption (current or within proceeding 2 months, > 2 drinks per day), current participation in regular exercise sessions ("Do you currently walk for at least 30-minutes at a time more than 1 time a week?"), or were currently participating in a research study.

**Figure 1:**
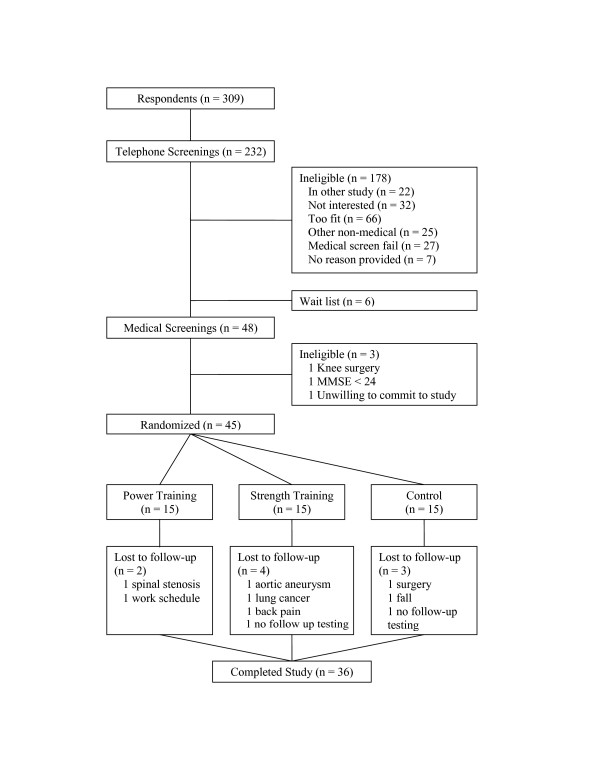
Participant Flow Diagram.

A brief pre-screening questionnaire to assess demographics, health status, and self-reported disability was administered to all respondents via telephone. Persons who passed the pre-screening were invited to the laboratory for a detailed screening for health and self-reported disability. Respondents meeting the eligibility criteria, who received written approval from their primary care physician, and who passed a resistance training safety screening (see below), were considered eligible for the study. The flow of participants through the study is illustrated in Figure 1. This study was approved by the university IRB and all participants gave written informed consent.

### Measures

#### Self-efficacy for Strength (SE)

Self-efficacy for strength was assessed in a hierarchical manner consistent with Bandura's [24] recommendations. Participants rated their confidence in their ability to successfully complete ten repetitions of increasing amounts of weight (5 items, 10 pounds up to 50 pounds in 10 pound increments). Items were scored on a scale of 0 to 100 and then averaged across the five items. Higher scores reflected greater self-efficacy for strength. This methodology has been used in numerous exercise studies and has been shown to be a valid and reliable method for assessing self-efficacy [25]. This scale demonstrated adequate reliability in the present study with Crobach alpha's of .82 and .92 at baseline and follow up, respectively.

#### Satisfaction with Physical Function (SPF)

Participants rated their satisfaction with six aspects of general physical function (e.g., level of physical fitness, level of endurance or stamina, physical ability to do what you want or need to do) on a 7-point scale ranging from +3 to -3: -3 (very dissatisfied), -2 (somewhat dissatisfied), -1 (a little dissatisfied), 0 (neutral), +1 (a little satisfied), +2 (somewhat satisfied), and +3 (very satisfied) [21]. Items were summed and averaged to obtain a total SPF score. This scale has been found to possess adequate psychometric properties [21]. This scale demonstrated adequate internal consistency in the present study (α's = .91-.94).

#### Life Satisfaction

The Satisfaction With Life Scale (SWLS) [20] was used to assess global quality of life. This measure was designed to assess an individual's global judgment of life satisfaction by allowing the respondent to weight the importance of life domains in accordance with his or her own values. The SWLS is a 5-item scale with each scale item rated on a 7-point scale ranging from 1 (strongly disagree) to 7 (strongly agree), with higher scores representing greater life satisfaction. Reviews of research using the SWLS suggest that it is sensitive enough to detect changes in life satisfaction over time [26]. The SWLS has adequate internal consistency and validity in research on aged populations and has been found to be responsive to physical activity in older adults [17,27]. The SWLS demonstrated adequate internal consistency in the present study (α's = .84-.88).

### Procedures

As a safety precaution, all participants completed a resistance training screening consisting of a series of lower extremity resistance exercises while heart rate (12 lead ECG), blood pressure, and symptoms were monitored. The data were reviewed by a physician who looked for abnormalities in heart rate and blood pressure. No participants were excluded based on the results of this screening procedure.

### Data Collection

Participants attended a baseline data collection visit in our laboratory in which basic demographic and biometric variables were assessed. In addition to age, gender, and education level, participants were assessed for body mass and height. Body Mass Index (BMI) was calculated using values of body mass and height. Muscle power and strength were assessed using pneumatic leg press (LP) and knee extension (KE) machines (Keiser Sports Health Equipment, Fresno, CA). These machines were also used for all power and strength training. The protocols and validation have been described previously [28]. Data collectors were not blinded to treatment arm allocation.

### PT and ST Interventions

The PT and ST interventions were conducted on Keiser pneumatic resistance machines that allowed fast and slow movement speeds without the risk of dropping weights or weight stacks. Training occurred at a central location and all sessions were supervised by an unblinded, trained interventionist. All participants attended an orientation session where the equipment and exercises were demonstrated. Participants trained in small groups (7–8 individuals) and rotated through the exercises stations. Participants in the power training group trained at a separate time than the strength training group. Although the focus of the intervention was the lower extremity, we included several upper body ST exercises using dumbbells (chest press, bicep curl, tricep kickback) and Nautilus machines (overhead shoulder raise, seated row). Participants completed three training sessions per week for 12 weeks. In each session participants completed three sets of 8–10 repetitions on the bilateral leg press (LP) and knee extension (KE) machines at 70% of their 1RM. The resistance on the machines was adjusted every two weeks by repeating the 1RM testing. The exercise interventionist monitored adherence and compliance with the protocol.

At the start of each training session all participants completed a supervised brisk walk or cycle ergometer ride of 10 min and a standard whole body muscle stretching routine. The interventionist made sure that participants adjusted the equipment appropriate to their body size and completed the resistance exercises with correct form. Training sessions ended with a 10 min stretching session.

Participants in the PT intervention were instructed to complete the concentric phase of the movement "as fast as possible", pause briefly at the midpoint of the movement and complete the eccentric phase of the movement in approximately 2–3 seconds. Participants in the ST intervention were instructed to complete the concentric phase of the movement in approximately 2–3 seconds, pause briefly at the midpoint of the movement and complete the eccentric phase of the movement in approximately 2–3 seconds.

Participants in the wait list control group were contacted each month to determine if they have made any significant changes in their lifestyle. In addition, as an incentive to participate, the control group was offered the more effective of the exercise interventions following the post-testing.

### Data Analyses

Separate ANCOVA's were conducted for each outcome variable which controlled for the baseline value and used the change from baseline to follow up as the dependent variable. Significant results were followed up with pairwise comparisons (with Bonferroni adjustments) and effect sizes were calculated. A p value of less than or equal to 0.05 was considered significant in all analyses.

## Results

Descriptive statistics provided in Table 1 illustrate that the three groups were essentially identical in terms of age, BMI, education, and distribution of men and women at baseline. In total, 15 participants were randomized to each of the three groups. At the 12-wk assessment, 12 PT, 11 ST, and 13 control group participants completed strength or power testing. Intervention adherence (defined as the mean of the participants' percent attendance and participation in the intervention) was 78% in the PT group and 64% in the ST group. Excluding participants who did not complete the final wk of the 12-wk intervention, adherence was 90% in the PT group and 79% for the ST group. We observed significant increases in strength for knee extension and leg press in both the PT and ST groups. Both groups also significantly increased lower extremity muscle power. However, there was a significantly greater increase in lower extremity muscle power in the PT group compared to the ST group (Marsh et al., In review).

**Table 1:** Participant Demographic Characteristics

	**Power Training (N = 15)**		**Strength Training (N = 15)**		**Control Group (N = 15)**		
	
	**Completers (N = 12)**	**Drop-Outs (N = 3)**	**Completers (N = 11)**	**Drop-Outs (N = 5)**	**Completers (N = 13)**	**Drop-Outs (N = 2)**	**P-Value For Completers**
**Age (yrs)**	76.8 (6.5)	78.0 (2.7)	74.1 (5.5)	73.0 (6.2)	74.3 (5.4)	70.0 (5.0)	0.47
**Gender (% female)**	46.7	20.0	66.7	20.0	53.3	20.0	0.54
**Height (cm)**	162.3 (10.0)	159.3 (8.1)	161.6 (13.1)	156.3 (4.9)	162.6 (8.8)	162.0 (7.2)	0.98
**Body Mass (kg)**	81.2 (18.3)	78.2 (14.1)	76.8 (16.9)	70.2 (20.3)	81.7 (21.0)	76.7 (8.1)	0.78
**Body mass index (kg/m**^2^**)**	30.7 (5.4)	30.8 (5.0)	29.5 (6.0)	28.4 (6.5)	30.8 (7.2)	29.4 (4.3)	0.85
**Education (no./%)**							0.83
HS or < HS	2 (13.3)	0	2 (13.3)	0	1 (6.7)	0	
>HS but <College	4 (26.7)	3 (20.0)	3 (20.0)	3 (20.0)	6 (40.0)	2 (13.3)	
> = College	6 (40.0)	0	7 (46.7)	0	5 (33.3)	1 (6.7)	

Note: * = independent sample t-test for differences between completers and drop outs; HS = High School

**Figure 2:**
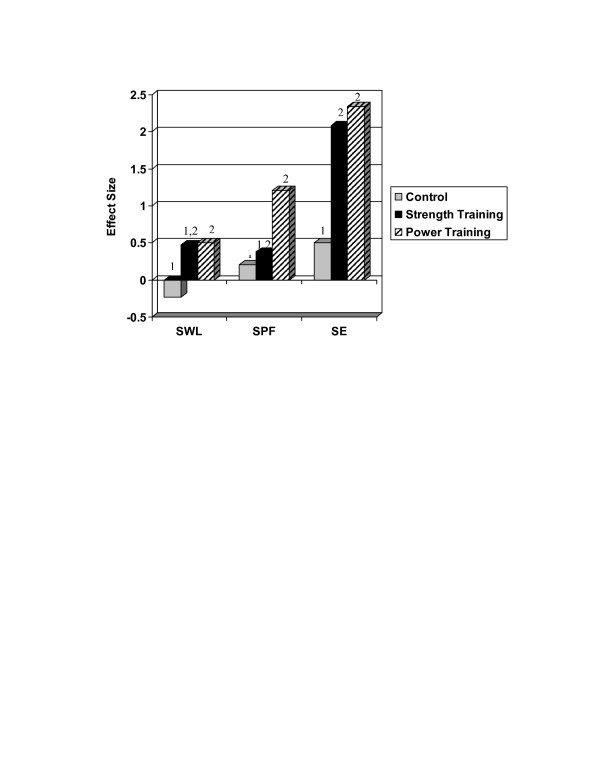
**Effect sizes for pre to post intervention changes in subjective well-being outcomes**. Note: SWL = Satisfaction With Life; SPF = Satisfaction with Physical Function; SE = Self-efficacy. Conditions not sharing a common superscript have significantly different change at p < .05.

Tables 2, 3, 4 provide descriptive statistics for self-efficacy, satisfaction with physical function, and satisfaction with life both at baseline and at the 3-month follow-up visit. In addition, the final column of each table provides the raw and baseline adjusted difference scores for each of the three treatment groups. Figure 2 illustrates the effect sizes across each of the experimental conditions for each outcome variable (ES = (M_post _- M_pre_)/SD_pooled_). One-way ANOVA's indicated that there were no significant between group differences on any of the outcome variables at baseline.

**Table 2:** Changes in Self Efficacy Across Treatment Conditions*

Treatment	Baseline: M ± SD	Post-Test: M ± SD	ΔScores: Raw (Adjusted M ± SE)
Control	25.07 ± 17.67	34.46 ± 19.88	9.39 (10.59 ± 6.14)^1^
ST	24.53 ± 14.68	65.00 ± 24.31	40.47 (41.41 ± 7.01)^2,a^
PT	25.07 ± 17.53	72.16 ± 22.58	47.09 (47.17 ± 6.43)^2,a^

ST = Strength Training, PT = Power Training

* = Baseline adjusted means in the last column not sharing a common superscript differ at the p < .05 level using a Bonferroni post hoc adjustment.

^a ^= significant within group change at p < .05 (paired t-test)

**Table 3:** Changes in Satisfaction with Function Across Treatment Conditions*

Treatment	Baseline: M ± SD	Post-Test: M ± SD	ΔScores: Raw (Adjusted M ± SE)
Control	-0.73 ± 1.69	-0.35 ± 1.96	0.38 (0.40 ± .38)^1^
ST	-0.44 ± 1.52	0.17 ± 1.71	0.61 (0.74 ± .42)^1,2^
PT	-0.85 ± 1.86	1.10 ± 1.20	1.85 (1.79 ± .40)^2,a^

ST = Strength Training, PT = Power Training

* = Baseline adjusted means in the last column not sharing a common superscript differ at the p < .05 level using a Bonferroni post hoc adjustment.

^a ^= significant within group change at p < .05 (paired t-test)

**Table 4:** Changes in Satisfaction with Life Across Treatment Conditions*

Treatment	Baseline: M ± SD	Post-Test: M ± SD	ΔScores: Raw (Adjusted M ± SE)
Control	22.93 ± 6.46	21.46 ± 6.06	-1.47 (-2.17 ± 1.46)^1^
ST	22.28 ± 6.79	25.27 ± 6.03	2.99 (2.38 ± 1.61)^1,2^
PT	25.78 ± 7.47	29.25 ± 6.38	3.47 (4.27 ± 1.62)^2,a^

ST = Strength Training, PT = Power Training

* = Baseline adjusted means in the last column not sharing a common superscript differ at the p < .05 level using a Bonferroni post hoc adjustment.

^a ^= significant within group change at p < .05 (paired t-test)

The series of ANCOVA's revealed significant group differences in changes in self-efficacy (F(2,31) = 9.77; p < .001), satisfaction with function (F(2, 32) = 3.36; p < .05), and satisfaction with life (F(2, 31) = 4.76; p < .05). Post-hoc analyses indicated that both the PT and ST groups experienced significant changes in self-efficacy (ES = 2.34 [PT]; 2.08 [ST]) as compared to the control group (ES = .5; p's <.05), but the amount of change did not differ between the two forms of resistance training.

Interestingly, in the case of satisfaction with physical function, the PT group (ES = 1.21) reported significantly more improvement than the control group (ES = .21) (p < .05). However, the amount of change in the ST group (ES = .38) did not differ from the control group and the amount of change in SPF did not differ between PT and ST. Additionally, follow up analyses indicated that although the PT group (ES = .5) experienced change in SWL that was significantly different than the control group (ES = -.23) (p < .05), the ST group change (ES = .47) was not significantly different than the control group. As with SPF, the change in SWL was not significantly different between ST and PT. The effect sizes across conditions for each outcome are displayed in Figure 2.

## Discussion

The purpose of the present study was to compare the influence of 2 different types of resistance training and a control on changes in measures of quality of life and self-efficacy in older adults. The data indicate that the mode of training resulted in a differential pattern of change. Whereas the PT condition resulted in increases in self-efficacy, satisfaction with physical function, and life satisfaction that were significantly different from a control condition of no exercise, ST differed from the control group only in changes in self-efficacy.

What is particularly noteworthy is that the PT condition produced dramatic effects across all measures after a relatively short intervention (12 weeks). As can be seen in Figure 2, the effect sizes for the PT condition ranged from 0.5 (SWL) to 2.34 (SE). Whereas we expected the behaviorally specific assessment of self-efficacy to be more sensitive to treatment, SWL represents a global judgment of one's life and is relatively stable [29]. That is, even significant life events, such as unemployment [30] and widowhood [29,31], have been found to have only small effects on life satisfaction. The existing research examining the influence of physical activity interventions on SWL typically involve aerobic exercise programs lasting at least 6 months [27,32]. For example, Fisher and Li [32] documented significant changes in SWL following a 6-month community walking program, but the effect size was .24. To our knowledge, our study is the shortest physical activity intervention, as well as the only resistance training study, to document significant changes SWL in older adults. It is possible that the findings reported here are simply short term benefits resulting from the novelty of participating in a unique strength training research study and that they will dissipate given longer a follow up assessment time frame. It is also possible that, due to our small sample, the findings are unique to this study. However, some studies have demonstrated that high velocity power training is more effective at improving physical functioning than traditional strength training [12]. Furthermore, lower physical functioning in valued activities produces dissatisfaction in older adults [33]. Therefore, it is also possible that the participants in the PT condition experienced greater benefits in physical functioning and subsequent improvements in satisfaction that were distinct and unique to this type of training. Clearly further research is needed to replicate and extend these findings.

This line of reasoning is supported by the differential effects observed on SPF and demonstrates important differences between the two resistance training conditions. Whereas participants in the PT condition experienced a large change in SPF (ES = 1.21), the ST condition produced only a small change (ES = .38). This finding is also important because decreased satisfaction with physical abilities is associated with greater physical impairment [34], greater disability with valued activities [35,36], and depressive symptoms [35]. Additionally, SPF has been found to mediate the influence of changes in physical activity participation on improvements in quality of life [37]. Thus, PT appears to be an effective intervention for increasing older adults' satisfaction with their physical functioning and these changes may have important implications for their overall quality of life.

The improvements in self-efficacy documented in the present study are consistent with a number of resistance training interventions [13,38-40]. What remains to be determined, however, is to what extent improvements in self-efficacy resulting from resistance training influence other health-related outcomes. For example, several studies have found self-efficacy to be a significant predictor of adherence to physical activity programs [41] as well as the long term maintenance of physical activity [42] in older adults. Additionally, self-efficacy has been found to predict the performance of several physical functions and mediate the influence of aerobic physical activity on stair climb performance in patients with knee osteoarthritis [43]. Furthermore, increases in self-efficacy resulting from a physical activity program have been shown to mediate the influence of physical activity on improvements in Satisfaction With Life [17]. However, these relationships have been examined in the context of aerobic physical activity (e.g., walking) and little is known regarding the influence of increases in self-efficacy related to strength and power on other health-related outcomes.

The results of the present study must be interpreted in the context of several study design limitations. As noted, the sample size was relatively small which resulted in low statistical power and limited the analyses. For example, although the ST condition resulted in change in SWL with an effect size of .47, these values were not statistically different from the control group. This null finding is most likely due to a lack of statistical power. We were also unable to examine the influence of potential moderators (e.g., gender). Additionally, the present study was not powered to examine structural pathways among the hierarchical assessment of quality of life. It would be of interest to determine the degree to which improvements in self-efficacy and SPF influence improvements of SWL. Conceptually, one might expect improvements at the activity specific level (self-efficacy) to lead to improvements at the domain level (satisfaction with physical function) which would lead to improvements at the global level (satisfaction with life). Finally, this study involved volunteer participants from the community, excluded non-completers from the analyses, and required strength training three days per week at our center using specialized strength training equipment. Therefore, it is possible the effects were influenced by the nature of the sample and it is not known whether these findings can be generalized to larger samples in community-based physical activity settings. It should also be noted that neither the participants nor the instructors were masked to treatment allocation.

## Conclusion

In conclusion, although both traditional strength training and high velocity power training enhance self-efficacy, power training may offer older adults unique benefits to multiple levels of quality of life beyond the influence of traditional strength training. Clinically, PT may be a particularly beneficial mode of training for older adults at risk for mobility disability [44] or other strength/muscle related disorders and lead to enhanced quality of life. Further research is needed that utilizes larger sample sizes to determine precisely why older adults' perceive PT differently than ST. Additionally, future research should examine the relationships between the improvements in quality of life found in the present study and any physiological and physical function changes that result from resistance training.

## Abbreviations

PT: Power Training; ST: Strength Training; SE: Self-efficacy; SPF: Satisfaction with Physical Function; SWL: Satisfaction with Life

## Competing interests

The authors declare that they have no competing interests.

## Authors' contributions

JAK participated in the data analysis and drafted the manuscript, WJR participated in the study design, data analysis, and helped to draft the manuscript, APM participated in the study design and coordination and helped draft the manuscript. All authors read and approved the final manuscript.
